# Delayed Diagnosis of Cystic Fibrosis and Nontuberculous Mycobacterial Infection in Refractory CRSwNP

**DOI:** 10.1002/rcr2.70681

**Published:** 2026-07-09

**Authors:** Robert Greig, Philipp Suter, Rory Chan, David Connell, Brian Lipworth

**Affiliations:** ^1^ Scottish Centre for Respiratory Research University of Dundee Dundee UK; ^2^ Ninewells Respiratory Department NHS Tayside Dundee UK

**Keywords:** chronic rhinosinositus, cystic fibrosis, mycobacterium abcessus, type 2 inflammation

## Abstract

Cystic fibrosis is a systemic disease inherited in an autosomal recessive pattern associated with multisystem pathology including increased mucus viscosity and consequently reduced clearance. Here we report a case of a 31 year old woman who experienced several years of treatment‐resistant type 2 low chronic rhinosinusitis. Repeat chest imaging revealed tree‐in‐bud change leading to a diagnosis of *Mycobacterium abscessus* and ultimately cystic fibrosis. She experienced a significant improvement following condensing *M. abscessus* eradication therapy and Kaftrio. This case outlines the importance of both considering atypical infection in treatment‐resistant chronic rhinosinusitis and the underlying pathology such as cystic fibrosis.

## Graphical abstract

This case describes a 31‐year‐old woman with treatment‐resistant sinus disease in which a delayed diagnosis of CF led to lung complications. It emphasises the importance of considering underlying causes like CF in unexplained, treatment‐resistant cases of chronic rhinosinusitis.

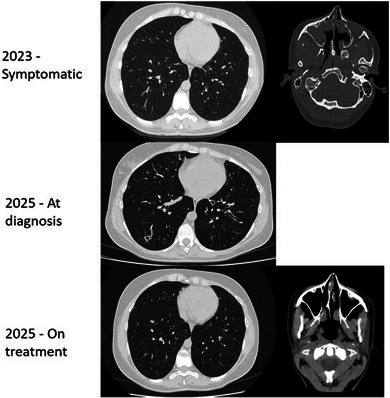

## 1 Introduction

Cystic fibrosis (CF) is a systemic disease inherited in an autosomal recessive pattern. It is characterised by impaired chloride ion transport across cells, which reduces water crossing the epithelium, resulting in increased mucus viscosity and consequently reduced clearance [1]. Dominant features of CF are recurrent respiratory infections and pancreatic insufficiency; chronic rhinosinusitis with nasal polyposis (CRSwNP) is also a commonly described symptom [2]. Viscous mucus within the airways can act as a reservoir for pathogens, thus resulting in recurrent infections.

## 2 Case Report

A woman in her 30s was referred to ENT with chronic rhinosinusitis with nasal polyposis (CRSwNP) and no otherwise significant medical history (year 0). She received oral corticosteroids (OCS) and antibiotics alongside intranasal corticosteroids with a CT sinus showing pansinusitis with obstruction of all the drainage pathways at that time. She underwent endoscopic sinus surgery the following year (year 1) due to poor treatment response. She continued to experience recurring upper respiratory tract infections indicating additional courses of OCS and antibiotics. Symptoms persisted with copious volumes of nasal discharge reported. Her 22‐item Sino‐Nasal Outcome Test (SNOT‐22), which measures the impact of nasal symptoms on quality of life where a score greater than 50 is reflective of severe disease, was 72/110. Between year 0 and year 3, blood analysis revealed normal white cell and eosinophil counts and immunoglobulins and a mildly raised C‐reactive protein (10–32 mg/L). Nasal secretion culture yielded no bacterial growth. Chest radiograph was normal. Histopathological examination of sinus biopsies showed mixed inflammatory infiltrates including eosinophils. At this point, she reported nocturnal coughing with occasional wheeze; thus budesonide/formoterol 200/6 μg was initiated for concurrent asthma. A sinus and high‐resolution chest CT reported pansinusitis with extensive bilateral sinonasal polyposis and no significant bronchiectasis.

**FIGURE 1:**
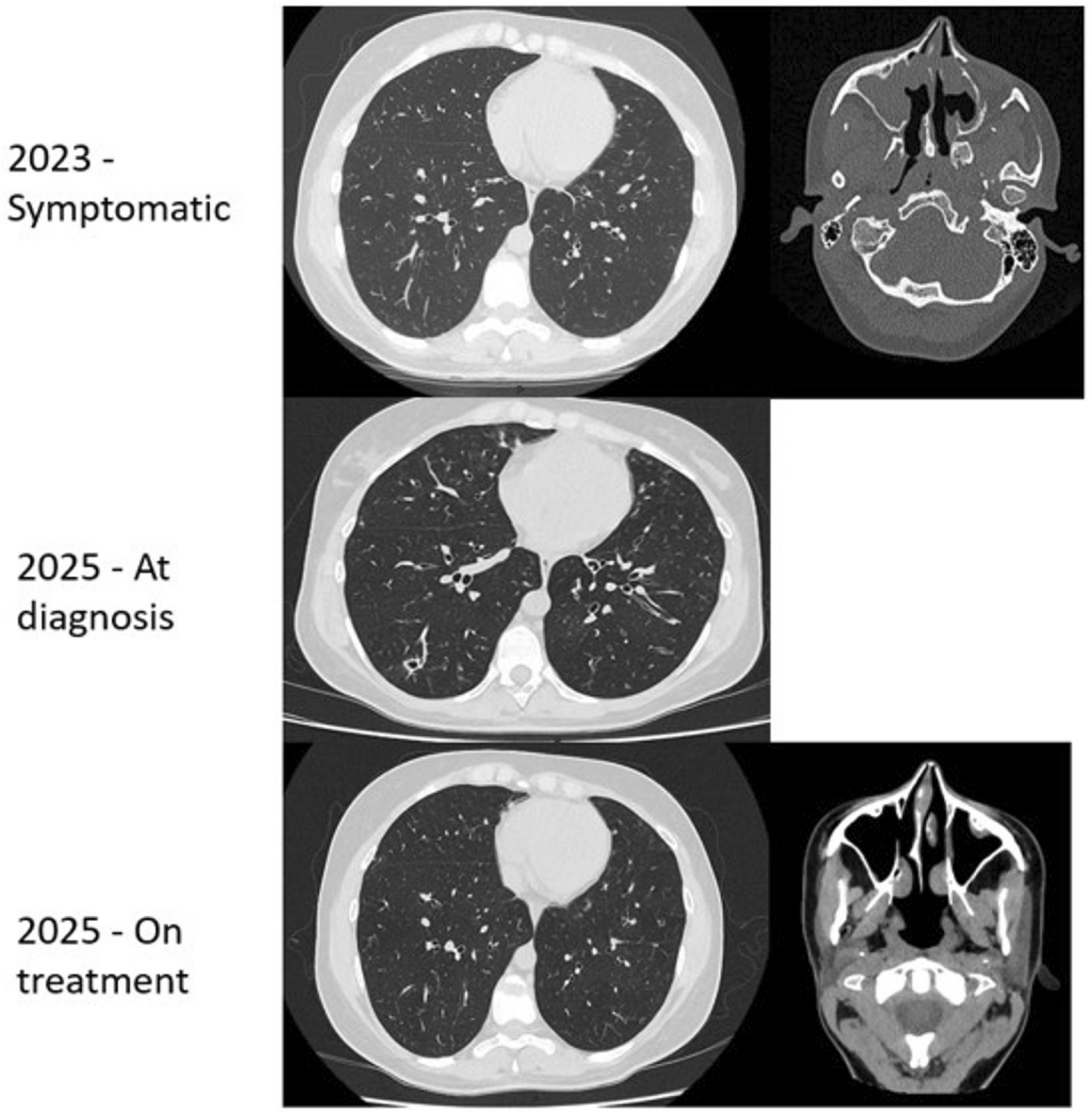
CT sinus and chest imaging prior, at time of *M. abscessus* diagnosis and once established on treatment.

In year 4, her symptoms remained unresolved with the addition of frequent lower respiratory tract infections. Her blood eosinophil count, total and specific IgE, and FeNO were normal, and rigid nasal endoscopy revealed large volumes of purulent secretions arising from the ostioemeal complex drainage. Pulmonary function tests (PFTs) revealed a decline in FEV_1_% predicted from 73% to 58% year 3 to year 5 respectively. Contrary to the initial report, reassessment of the chest CT identified early bronchiectasis with tree‐in‐bud (Figure 1). Sputum samples were positive for *Mycobacterium abscessus* . A repeated chest CT showed bilateral cavitating lesions and pulmonary nodules with tree‐in‐bud (Figure 1).

Following this, referral was made to the pulmonology infection service, where further history revealed drenching night sweats for 3 years and abdominal discomfort with steatorrhea. She had a positive sweat test, and genetic testing was consistent with CF transmembrane conductance regulator (CFTR) related disease, heterozygous for the pathogenic CFTR variant c.1652G>A (p.Gly551Asp) and for the intronic variants (TG)12T5 and (TG)10T7. She was admitted for *M. abscessus* eradication with 4 weeks of IV Tigecycline, Amikacin and Imipenem/Cilastatin and followed by a further 17 months of oral azithromycin, clofazimine and nebulised amikacin. She was also started on Elexacaftor/Tezacaftor/Ivacaftor (Kaftrio). On recent follow up (year 5), she reported symptom improvements with her SNOT‐22 score dropping to 26 and nasal endoscopy showing normal mucosa with a grade 1 polyp. FEV1% predicted increased to 84%. On repeat imaging nodular opacities improved; however, some tree‐in‐bud persisted (Figure 1).

## 3 Discussion

Chronic rhinosinusitis with nasal polyposis (CRSwNP) is a common presentation to the ENT clinic. However, in this case, we encountered a young patient with progressive symptoms resistant to treatment. Initial cultures did not include mycobacterium and therefore yielded no growth. Potential delay in diagnosis resulted from initial focus on nasal symptoms without additional exploration of her chest complaints. There is significant symptom and severity variation within CF based on the underlying CFTR gene variant [3]. However, the association of CF and CRSwNP is well recognised, with the most common complaints being nasal congestion and purulent discharge which can represent infection [2]. The association of CF with pulmonary *M. abscessus* infection is also well recognised [4]; however, mycobacterium rhinosinusitis is significantly less common and tends to be associated with immune dysfunction and previous chemoradiation which our patient did not have [5]. Treatment and eradication of *M. abscessus* is challenging. It requires a prolonged regimen of multiple medications with a substantial adverse effect burden. Despite this, eradication is only achieved in 28%, with 87% of patients experiencing at least one side effect [4]. Our case highlights that in patients with CRSwNP, particularly those who are young with low type 2 biomarkers and no apparent risk factors, more atypical causes should be considered. Pointedly, in unexplained mycobacterium infection, CF should be considered due to the critical role the CFTR gene plays in defence against *M. abscessus* [6]. It took 4 years from presentation to diagnosis allowing significant pulmonary disease to develop. This time could have been a missed opportunity to identify the underlying pathology and initiate treatment.

In conclusion, CRSwNP is a common presentation to the ENT clinic. However, treatment resistant disease with recurrent infections and purulent discharge should prompt consideration of more atypical causes.

## Author Contributions

All authors (R.G., P.S., R.C., D.C. and B.J.L.) were involved equally in concept, writing and editing.

## Funding

The authors have nothing to report.

## Consent

The authors declare that written informed consent was obtained for the publication of this manuscript and accompanying images and attest that the form used to obtain consent from the patient(s) complies with the Journal requirements as outlined in the author guidelines.

## Conflicts of Interest

Dr. Greig reports personal fees (talks) from AstraZeneca. Dr. Suter reports personal fees (talks) from AstraZeneca, personal fees (talks) from GSK, grants from Lung League Fribourg (Switzerland), grants from Swiss Lung Foundation (Switzerland). Dr. Chan reports institutional grants awarded from Asthma+Lung UK, Chiesi, AstraZeneca and GSK; serving on advisory boards for AstraZeneca and Vitalograph; personal fees (talks and/or drafting educational material) from AstraZeneca, Chiesi, Thorasys and Vitalograph; and support attending meetings from AstraZeneca, Chiesi, NIOX, Sanofi‐Regeneron and Vitalograph. Dr. Connell reports personal fees (talks and consulting) from AstraZeneca, personal fees (talks and consulting) from GSK, and personal fees (talks and consulting) from Insmed. Dr. Lipworth reports non‐financial support (equipment) from GSK; grants, personal fees (consulting, talks and advisory board), other support (attending ATS and ERS) and from AstraZeneca; personal fees (talks and consulting) from Sanofi, personal fees (consulting, talks and advisory board) from Circassia in relation to the submitted work; grants, personal fees (consulting, talks, advisory board), other support (attending ERS) from Teva, personal fees (talks and consulting), grants and other support (attending ERS and BTS) from Chiesi, personal fees (consulting) from Lupin, personal fees (consulting) from Glenmark, personal fees (consulting) from Dr. Reddy, personal fees (consulting) from Sandoz; grants, personal fees (consulting, talks, advisory board), other support (attending BTS) from Boehringer Ingelheim, grants and personal fees (advisory board and talks) from Mylan outside of the submitted work; and the son of BJL is presently an employee of AstraZeneca.

## Data Availability

The data that support the findings of this study are available on request from the corresponding author. The data are not publicly available due to privacy or ethical restrictions.
